# Holobiontic Intercellular Relationships Between the Oral Cavity and the Rest of the Human Organism: A Narrative Review

**DOI:** 10.3390/medicina62071365

**Published:** 2026-07-15

**Authors:** Vasile Burlui, Daniela Luminița Ichim, Daniela Ivona Tomița, Malina Visternicu, Alin Ciobica, Mihaela Diana Gheban

**Affiliations:** 1 Preclinical Department, Apollonia University, Păcurari Street 11, 700511 Iași, Romania; secretariat@univapollonia.ro (V.B.); diana.gheban@yahoo.com (M.D.G.); 2 “Ioan Haulica” Institute, Apollonia University, Păcurari Street 11, 700511 Iași, Romania; danielaluminitaichim@yahoo.com (D.L.I.); malina.visternicu@yahoo.ro (M.V.); alin.ciobica@uaic.ro (A.C.); 3 Doctoral School of Biology, Faculty of Biology, “Alexandru Ioan Cuza” University of Iasi, Carol I Avenue, 20A, 700505 Iași, Romania; 4 Department of Biology, Faculty of Biology, “Alexandru Ioan Cuza” University of Iasi, Carol I Avenue, 20A, 700505 Iași, Romania; 5 “Olga Necrasov” Center, Department of Biomedical Research, Romanian Academy, 700481 Iași, Romania

**Keywords:** holobiont, hologenome, oral microbiota, oral dysbiosis, inflammation

## Abstract

The holobiont represents a fundamental concept in modern biology, defining the organism as a complex unit composed of the host and its symbionts (microbes, viruses) that live together, forming an integrated biological system in which the host and microbes collaborate and influence each other (genetically and metabolically) and evolve as a single entity, rather than the host evolving in isolation. It is recognized that the health and functioning of the host fundamentally depend on its microbiome, consolidating the entire assembly as a unit of evolutionary selection with a shared genome called the hologenome. The oral microbiota plays an essential role in maintaining homeostasis and in modulating epigenetic processes, having unique characteristics due to the oral environment and microbial diversity. The aim of this narrative review is to explore how the oral microbiota interacts with host cells through microbial metabolites, including short-chain fatty acids (SCFAs), microRNAs (miRNAs), extracellular vesicles, and cellular signaling pathways, influencing prenatal and perinatal development as well as overall health. By critically integrating current evidence, this narrative review provides an updated conceptual framework linking the oral microbiota, the holobiont concept, epigenetic regulation, and prenatal and postnatal life. It advances the interpretation of the current literature by bringing together molecular, immunological, and developmental mechanisms that are commonly discussed separately, highlighting the oral microbiota as an active epigenetic regulator within the human holobiont. The effects of oral dysbiosis on both local and systemic health are analyzed, including inflammatory responses, periodontal health, and the risk of chronic diseases or cancer. In addition, the importance of maintaining microbiome homeostasis starting from the gestational period is discussed, in order to prevent epigenetic disturbances that may affect fetal development and postnatal oral health. Collectively, the available evidence supports the biological relevance of the oral holobiont in health and disease while highlighting its potential clinical implications. However, further mechanistic and longitudinal studies are needed to validate these associations and to clarify the causal pathway underlying host–microbiota interactions.

## 1. Introduction

In biological and ecological research, the close relationship between the host and its symbionts is considered a fundamental concept in modern biology [1]. The human organism, and particularly the oral cavity, has served since the early stages of species evolution as a host for a diverse and abundant microbiota. The study of the relationships between the host and its microbiota has evolved significantly in recent decades, focusing on how microbial communities influence the health and development of the organism. In particular, the oral cavity represents a unique ecosystem characterized by extremely high microbial diversity, which constantly interacts with host tissues and contributes to the regulation of both local and systemic biological mechanisms [2,3,4].

In this context, the microbiota can no longer be regarded merely as a simple collection of microorganisms, as it functions as a true “ecological organ”, capable of modifying immune responses, metabolic processes, and even gene expression through epigenetic mechanisms, thereby influencing the evolution and adaptation of the host [5,6]. Microbiota-sensitive epigenetic modifications include alterations of DNA or histones, as well as the regulation of non-coding RNAs [7]. Signals originating from the microbiota can be integrated at the level of host cells through epigenetic mechanisms that modify their functional programs without altering the underlying genetic sequence [7]. These mechanisms include DNA methylation, histone modifications, and the regulation of non-coding RNAs.

The prenatal and perinatal periods represent critical stages during which interaction with the microbiota may determine long-term biological programming, affecting the development of the immune system and susceptibility to various later-life conditions [8]. This perspective extends the holobiont concept by emphasizing that host–microbiota interaction begins before birth and continues throughout life, contributing to long-term immune and metabolic programming.

Moreover, the holobiontic approach offers an integrated view of how microbes collaborate with the host, not only by maintaining homeostasis but also by contributing to the regulation of inflammatory responses, tissue repair, and the prevention of chronic diseases [9]. The aim of this narrative review is to synthesize current evidence on the epigenetic interaction between the oral microbiota and the human host, with particular emphasis on microbial metabolites, extracellular vesicles, and miRNAs, and to discuss their relevance for oral health and systemic diseases, and early-life biological programming. This review synthesizes current evidence without adopting a systematic or quantitative approach and addresses the lack of an integrated framework connecting microbiota-derived signals (metabolites, extracellular vesicles, and miRNAs) with epigenetic mechanisms across oral and systemic systems.

## 2. Methodology

### 2.1. Search Strategy

This study was designed as a narrative review synthesizing current evidence on the interactions between the oral microbiota, the holobiont concept, and epigenetic regulation. A targeted literature search was performed using PubMed, Scopus, and Google Scholar to identify relevant publications. The literature search was performed up to May 2026. Only articles published in English were considered. The search strategy combined predefined keywords including: “Holobiont”, “Hologenome”, “Oral microbiota”, “Oral dysbiosis”, and “Inflammation”. Additional related terms such as “epigenetics”, “microbial metabolites”, and “miRNAs” were also used to broaden the scope of the search. Boolean operators (AND, OR) were used to combine search terms where appropriate. Reference lists of relevant articles were also manually screened to identify additional eligible publications.

### 2.2. Eligibility Criteria

Studies were included if they addressed the relationships between oral microbiota, host epigenetic regulation, and systemic or oral health within the framework of the holobiont concept. Priority was given to recent review articles, experimental studies, and clinically relevant publications addressing host–microbiota interactions and epigenetic mechanisms. When appropriate, landmark studies were also included to provide historical and conceptual context. Articles were excluded if they were non-peer-reviewed publications, case reports, conference abstracts, and editorials.

### 2.3. Study Selection and Data Synthesis

Following the literature search, relevant studies were screened based on titles and abstracts, and full texts were assessed for eligibility according to the inclusion and exclusion criteria. Data extracted from the selected studies included the study design, research focus, principal findings, and relevance to the objectives of this review. The evidence was subsequently synthesized narratively. To improve clarity and facilitate interpretation of the evidence, the main characteristics and findings of the included studies are summarized in Table 1. When appropriate, landmark studies were also included to provide historical and conceptual context.

## 3. Results

### 3.1. The Origin of the Concept of Symbiosis

The concept of symbiotism was developed and deepened by Lynn Margulis, who in 1991 emphasized the fundamental role of biological cooperation in the evolution of complex life forms. According to this perspective, in the early stages of evolution, following the formation of the universe, prokaryotic organisms first appeared. These primitive cells interacted and associated in various environments, and through processes of cooperation and biological integration they contributed to the emergence of eukaryotic cells, complex cellular structures characterized by the presence of a nucleus and numerous specialized organelles.

The first ideas regarding this concept were formulated by Margulis in an article written in 1966. However, the paper was initially rejected by 15 scientific journals before the theory was later recognized and accepted by the scientific community. Subsequently, additional research provided important evidence supporting this theory. Thus, in 1978, Randolph Schwartz demonstrated the origin of mitochondria from ancestral bacteria, while chloroplasts were identified as originating from cyanobacteria [17].

These discoveries led to the formulation of the hypotheses of endosymbiotic theory and eukaryotic evolution, which were further developed and consolidated in the 1980s. According to these theories, prokaryotic cells contributed to the formation of eukaryotic cells through processes of intercellular communication and through the exchange of biological material, including cellular components and genetic information [18]. From this evolutionary perspective, the emergence of complex biological organisms can be understood as the result of a long process of cooperation and integration between different forms of microscopic life. In this context, an essential question arises: if prokaryotic microorganisms formed the basis for the emergence of eukaryotic cells and, implicitly, of complex organisms, what are the consequences of the continuous cohabitation between the human organism and the vast microbial flora that inhabits it?

### 3.2. The Concept of Holobiont

The concept of the holobiont was formally developed in 2008 by Eugene Rosenberg and Ilana Zilber-Rosenberg, who redefined the way we perceive organisms and their ecological relationships, although the term „holobiont” has earlier histological references in the literature. About ten years later, the holobiont, composed of the host and its microbiome, came to be viewed as a single biological entity that functions at anatomical, metabolic, immunological, and evolutionary levels. The hologenome combines the stable genome of the host with the microbiome genome, which is dynamic and rapidly adaptable to the environment, providing an additional mechanism for genetic variation and natural selection [19].

The holobiont represents an ecological unit composed of a host and all its symbionts, including microbes, viruses, and fungi [12,20,21], highlighting that organisms do not exist in isolation but rather in close interdependence with the microbial communities that accompany them [22]. The host is the primary organism, and in humans the microbiota represents the totality of microbes that live in a specific area or throughout the entire organism [23,24]. It is estimated to consist of approximately 100 trillion cells, corresponding to roughly 5 kg of microbial mass.

The host genome represents the inherited genetic endowment, while the microgenome refers to the genetic endowment of the microbiota [25]. The hologenome represents the total genetic material of the host and its microbiota, that is, the entire holobiontic system, while the epigenome includes the genetic information that induces changes in gene expression without altering the DNA sequence [1,10]. The microbial component of the human holobiont contributes a vastly larger number of genes compared to the human genome. Current estimates suggest that microbial genes outnumber human genes by approximately 100:1 to 150:1. This comparison refers specifically to the diversity of microbial open reading frames present in the metagenome, rather than proportions of total genetic material, genomic mass, or DNA content [26]. The concept is grounded in the discoveries of James Watson and Francis Crick regarding the structure of DNA and its genetic role [27]. Understanding the holobiont and the hologenome changes the biological paradigm, highlighting the importance of complex relationships between the host and its microbiota for health and evolution.

The oral cavity represents a complex ecosystem that hosts a diverse microbiota including more than 700 bacterial species, as well as fungi, viruses, and protozoa [28]. Among the commensal species are *Streptococcus salivarius*, *Streptococcus mitis*, *Streptococcus sanguinis*, *Actinomyces naeslundii*, and *Veillonella parvula*, which contribute to maintaining microbial balance, immune protection, and microbial competitiveness [29,30]. In contrast, pathogenic species associated with oral diseases, such as *Streptococcus mutans*, *Porphyromonas gingivalis*, *Tannerella forsythia*, *Treponema denticola*, and *Fusobacterium nucleatum*, can cause dental and gingival disorders [31,32,33].

The microbiota colonizes different niches within the oral cavity, including the tooth surface, gingival sulcus, tongue, palate, and oral mucosa [34,35]. The oral cavity functions as a true “thermostat” favorable to the development of microorganisms due to its constant temperature, moisture, relatively anaerobic environment, darkness, the presence of suitable niches, and the nutritional support it provides [36].

### 3.3. Communication Between the Oral Cavity and the Rest of the Body

The oral cavity constantly communicates with the rest of the organism through its structures, including the labio-buccal and palatal mucosa, the periodontium, the tongue, and the salivary glands. These structures allow the transfer of signals and substances between the oral microenvironment and the tissues of the body. Intercellular communication in metazoans occurs through various mechanisms: cytoplasmic bridges, exosomes and ectosomes, interactions between membrane protein molecules, extracellular vesicles, and soluble messenger molecules [37,38].

These signals control the activity of cells located at a distance and include hormones, neurotransmitters, cytokines, growth factors, and morphogenic factors. The target cell may belong to the same organism or, in some cases, to another organism, even from different species or kingdoms [39,40]. The types of cellular communication that maintain homeostasis involve the endocrine system, through specific molecules; the nervous system, through neuronal impulses; the immune system, through specialized molecules; and possibly biophotons [41,42].

Communication involving oral structures occurs through two main pathways: between the cells of the oral cavity and the other cells of the organism, and between the cells of the oral cavity, the symbiotic agents of the microbiota, and the rest of the organism [2,28]. Thus, the oral cavity is not only a site of microbial colonization but also a center for the transmission of biological signals that influence the health and balance of the entire organism [13].

#### The Oral–Systemic Axis

Recent research has increasingly emphasized the existence of an “oral–systemic axis”, through which oral microorganisms and their metabolites influence distant organs and systems [43]. Oral bacteria may disseminate hematogenously or through the digestive tract, contributing to systemic inflammation and chronic diseases. Species such as *Porphyromonas gingivalis* and *Fusobacterium nucleatum* have been associated with cardiovascular diseases, colorectal cancer, neurodegenerative disorders, and metabolic syndromes [14,44,45]. These findings support the concept that the oral cavity acts not only as a local microbial ecosystem but also as a systemic biological interface within the human holobiont.

The mechanisms underlying the oral–systemic axis include translocation of microbial components such as lipopolysaccharides, activation of Toll-like receptor (TLR) signaling pathways, and subsequent stimulation of systemic inflammatory cascades mediated by cytokines including interleukin-6, tumor necrosis factor alpha, and C-reactive protein [46,47,48]. In addition, oral microbiota-derived extracellular vesicles and metabolites may modulate endothelial dysfunction, blood–brain barrier permeability, and metabolic homeostasis, thereby linking oral dysbiosis to distant organ pathology [49,50,51,52].

### 3.4. The Role of the Periodontium

The periodontium plays an essential role in the communication between the oral cavity and the rest of the organism. It is a complex histological structure composed of several types of tissues: epithelial, connective, ligamentous, bone tissue, root cementum, nerve fibers, and sensory receptors. It is served by a terminal arterial, venous, and lymphatic system [53,54,55].

These periodontal structures establish relationships of tissue partnership and local, regional, and holistic integration. They facilitate coordination between the periodontal cellular community and the entire organism through humoral, endocrine, nervous, informational, and mixed pathways. The periodontium acts as a complex system capable of receiving, processing, storing, and transmitting information to the body’s compartments, which can then take appropriate actions, according to the descriptions of Jim Crutchfield.

During the acute inflammatory response stage, the periodontium initiates the biological cascades necessary for repair and regeneration. This includes cellular infiltration, angiogenesis, formation of granulation tissue, re-epithelialization, and tissue remodeling [56,57]. Thus, the periodontium is not only a structure that supports the teeth but also an active component in maintaining the homeostasis and health of the organism.

**Figure 1:**
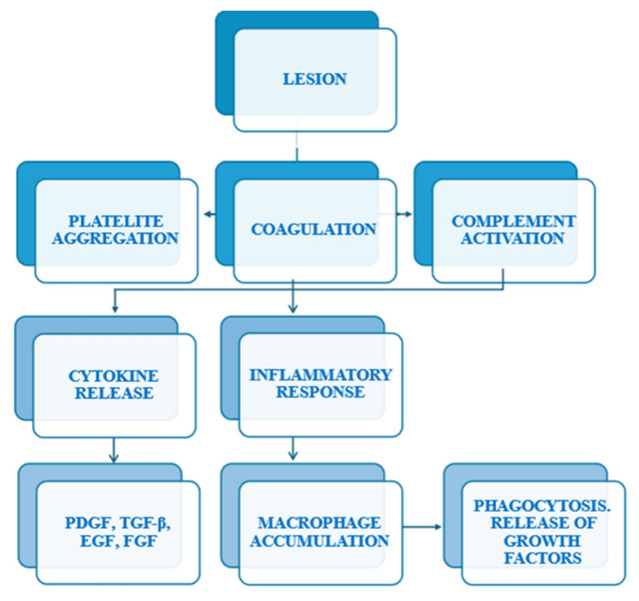
Biological processes that are triggered in response to tissue injury.

Figure 1 illustrates the biological processes that are triggered in response to tissue injury. Initially, the injury activates blood coagulation and the complement system, as well as platelet aggregation. These events lead to the release of cytokines and the initiation of an inflammatory response, which is essential for the recruitment of immune cells to the affected area.

The inflammatory response promotes the accumulation of macrophages, cells that eliminate dead cells and microorganisms and release growth factors such as platelet-derived growth factor, transforming growth factor beta, epidermal growth factor, and fibroblast growth factor. These factors stimulate tissue regeneration, angiogenesis, and local remodeling.

Thus, the response to injury involves a complex cooperation between coagulation, cellular signaling, activation of the immune system, and tissue regeneration. This coordinated interaction allows the restoration of the normal structure and function of the tissue, thereby maintaining the homeostasis of the organism.

In response to signals transmitted by mast cells, the biological processes that constitute the repair and healing cascade are triggered, including cellular infiltration, angiogenesis, granulation tissue formation, re-epithelialization, and tissue maturation and remodeling. All these stages are coordinated to restore the integrity and functionality of the affected tissues, demonstrating how the periodontal system and immune cells collaborate to maintain homeostasis and support the organism’s regeneration.

**Figure 2:**
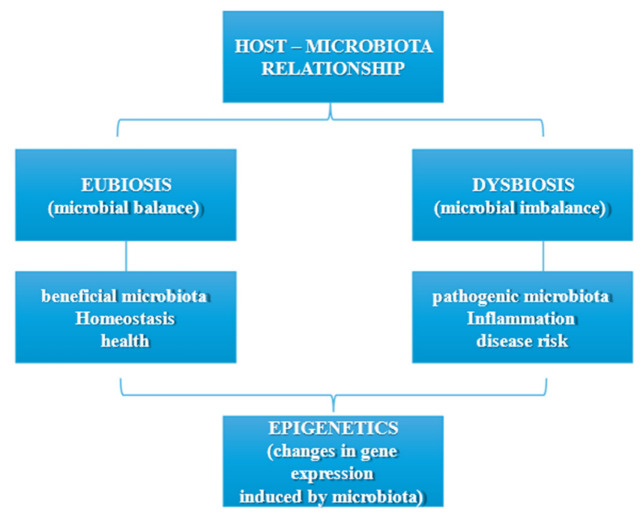
The host–microbiota relationship and its impact on health and epigenetics.

In addition to the role of mast cells and immune cells in tissue regeneration, the state of the oral microbiota plays a crucial role in maintaining homeostasis and modulating the inflammatory response. Figure 2 illustrates how the host–microbiota relationship, whether in eubiosis (microbial balance) or dysbiosis (microbial imbalance), can influence periodontal health and epigenetics, which are essential aspects for the processes of repair and healing.

### 3.5. Oral Dysbiosis

Communication between the cells of the oral cavity and their symbiotic agents, namely the microbiota, involves three main determinants. When dysbiosis occurs, the microbial balance of the oral cavity is disrupted, generating microbial aggression against oral structures, including the periodontium through marginal periodontitis, the tongue through glossitis, and the palatal or labio-buccal mucosa, the latter being affected much more rarely. Although these categories are studied in specialized clinical disciplines, dysbiosis is associated with the development of dental caries, periodontal diseases, and certain systemic conditions through inflammation and bacterial translocation.

Bacteria such as *Porphyromonas gingivalis* and *Fusobacterium nucleatum* can induce local epigenetic changes, periodontal disease, increased cancer risk, and systemic dissemination through migration or translocation. Periodontal disease remains a classic example of imbalance between the local microbiota and the host’s inflammatory response, and dysbiosis results from an inefficient and poorly controlled local inflammatory response in susceptible individuals [58,59,60].

This leads to inflammatory degradation of the periodontal system and consequences such as caries, periodontal disease, glossitis, stomatitis, or infections and abscesses in oral spaces, highlighting the importance of maintaining microbial balance and effective communication between the oral microbiota and the host immune response [59].

### 3.6. The Microbiota-Epigenetic Relationship

The relationship between the oral microbiota and epigenetics demonstrates how oral dysbiosis can increase the risk of epigenetic modifications [61]. Dysbiosis alters the epigenetic profile of local immune cells, which can migrate throughout the body carrying inflammatory signals [59]. The microbiome-epigenome axis influences gene expression through metabolites such as histone deacetylase inhibitors (HDACs) and DNA methylation [62,63]. This can induce hypermethylation of tumor suppressor genes in epithelial cells [64]. Epigenetic modifications, such as DNA methylation of tumor suppressor genes, promote cancer development [65].

Several oral pathogens have been shown to induce specific epigenetic alterations in host tissues. For example, *Porphyromonas gingivalis* promotes aberrant DNA methylation and histone modifications that alter the expression of genes involved in epithelial integrity, inflammatory signaling, and immune regulation. Similarly, *Fusobacterium nucleatum* has been associated with dysregulation of host microRNAs and activation of NF-κB signaling, thereby contributing to chronic inflammation and carcinogenesis. These findings demonstrate that oral dysbiosis influences host biology through precise molecular mechanisms rather than solely through direct bacterial invasion [66].

SCFAs are important microbial metabolites with recognized activity, particularly through inhibition of HDACs. While SCFAs have been extensively investigated in the intestinal microbiota, their biological role in the oral cavity differs substantially. In periodontal niches, SCFAs such as butyrate are produced primarily by proteolytic anaerobic bacteria, including *Porphyromonas gingivalis*, *Fusobacterium nucleatum*, and *Treponema denticola*, rather than by the fermentation of dietary fibers. Depending on their local concentration, these metabolites may exert immunomodulatory effects under physiological conditions but can also contribute to epithelial toxicity, apoptosis, and periodontal tissue destruction during oral dysbiosis. Therefore, the biological effects of SCFAs should be interpreted within the specific ecological context of the oral microbiome [7].

The reviewed evidence suggests that oral microorganisms may modulate host epigenetic and immune pathways through extracellular vesicles, quorum-sensing molecules, SCFAs, and activation of TLR signaling. These mechanisms influence inflammatory cascades such as nuclear factor kappa-light-chain-enhancer of activated B cells activation, cytokine production, and immune cell differentiation, thereby contributing to chronic inflammation and tissue remodeling [50,67].

DNA ensures the biological continuity of the species, storing information transmitted from parents to offspring and replicating faithfully to allow intercellular information transfer [68]. DNA methylation is a crucial epigenetic control mechanism in mammals, influencing gene expression by regulating transcription factor accessibility, histone modifications, and chromatin transcriptional activity. This modification is itself tightly regulated, involving components that add or remove methyl groups. DNA methyltransferases catalyze the transfer of a methyl group from the donor S-adenosylmethionine to the carbon-5 position of cytosine in DNA, producing 5-methylcytosine [15]. The main effect of DNA methylation is genetic silencing (gene inactivation without altering the sequence) and compaction (preventing enzyme access to the DNA), whereas histone acetylation acts like a zipper that opens DNA structure and facilitates gene activation [69].

Histones (H2A, H2B, H3, and H4) form the nucleosome core, which is wrapped by DNA. Multiple modifications can occur on these histones, including acetylation, methylation, phosphorylation, and ubiquitination, mainly on the N-terminal tails, controlling gene expression. Acetylation activates transcription, while deacetylation represses it. The microbiota influences these modifications through enzymes and metabolic products (e.g., SCFAs) [15].

Epigenetic regulation by the microbiota involves proinflammatory, immune, and metabolic reactions, including folates and vitamins B2 and B12, which donate methyl groups for DNA or histone methylation [7,70,71]. Epigenetic modifying enzymes require appropriate substrates to catalyze chromatin modifications. Epigenetic regulation activates enzymes such as methyltransferases and acetyltransferases and stimulates cellular mechanisms that direct epigenetic pathways [72]. DNA and histone methyltransferases, as well as histone acetyltransferases, commonly use methyl or acetyl donors to activate their catalytic activity [7].

Thus, the gut microbiota plays an important epigenetic role by inducing chronic inflammation, regulating gene expression, and providing essential metabolites such as short-chain fatty acids (butyrate, propionate, acetate) and folates [73,74]. This highlights the profound influence of microbial communities on local and systemic health through epigenetic mechanisms.

Although the intestinal microbiota has provided important insights into microbiota-mediated epigenetic regulation, the present review focuses primarily on the oral cavity. Nevertheless, the oral and gut microbiomes are interconnected through the oral-gut axis, whereby oral microorganisms are continuously swallowed and may ectopically colonize the gastrointestinal tract, particularly under conditions of dysbiosis. This process has been associated with alterations in gut microbial ecology, immune responses, and systemic inflammation. Consequently, findings from intestinal microbiome research are discussed here only as a comparative model that helps explain common epigenetic mechanisms underlying host–microbiota interactions [63,73,74,75,76,77].

### 3.7. The Lingual Microbiota as a Microbial Reservoir

The tongue represents a major ecological niche within the oral cavity and functions as a stable microbial reservoir. Its dorsal surface hosts a dense and diverse microbial community, which contributes significantly to overall oral homeostasis. In early life, particularly in infants under 18 months of age, the microbial load on the tongue can exceed that of dental surfaces, due to the limited presence of tooth enamel and the predominance of soft oral tissues [78]. This highlights the tongue as a primary site of microbial colonization during early oral development [76].

The lingual microbiota plays an important role in seeding and maintaining the overall oral microbial ecosystem [78]. Imbalances in this niche may contribute to dysbiosis and influence the risk of oral diseases such as dental caries and gingival inflammation, with potential systemic implications through microbial translocation and inflammatory pathways [4]. Mechanical removal of tongue biofilm has been shown in clinical studies to reduce salivary levels of cariogenic bacteria, particularly *Streptococcus mutans*, and to decrease overall plaque indices [78]. These findings suggest that tongue cleaning, when integrated into oral hygiene routines, may support the control of pathogenic microbial load and contribute to caries prevention in pediatric patients.

Furthermore, oral microbial dysbiosis has been increasingly associated with systemic conditions through mechanisms involving microbial translocation and host inflammatory responses [79]. Although evidence remains largely associative, the oral cavity, including the tongue as a microbial reservoir, may play a contributory role in broader host–microbiome interactions affecting systemic health [80].

### 3.8. The Role of miRNA

The role of miRNAs in the oral cavity and in the microbiota–host relationship is essential for epigenetic transmission and the fine regulation of gene expression. These miRNAs function by binding to RNA molecules and blocking their translation [81]. Signals from microbial non-coding RNA interact with the host’s miRNAs, which in turn contribute to maintaining epithelial integrity. Several specific miRNAs have been implicated in the oral–systemic axis. For example, miR-21 and miR-155 are consistently associated with chronic periodontal inflammation and immune dysregulation, whereas miR-146a has been linked to the modulation of inflammatory signaling pathways and host immune homeostasis. Dysbiosis-associated pathogens, particularly *Porphyromonas gingivalis*, have also been reported to induce epigenetic alterations, including aberrant DNA methylation of host genes involved in epithelial integrity and immune responses, further contributing to chronic inflammation and disease progression [82,83].

This highlights the complex way in which genetic and epigenetic elements, including microbial components, communicate and coordinate biological functions to maintain oral and systemic health.

#### Salivary Exosomes and Biomarkers

Saliva contains extracellular vesicles and exosomes that transport proteins, lipids, DNA fragments, and microRNAs involved in intercellular communication [37,84]. Salivary exosomes represent an important mechanism through which oral tissues and microbiota may influence distant organs. Due to their accessibility and biological content, salivary biomarkers are increasingly investigated for the early diagnosis of inflammatory, metabolic, and neoplastic diseases [85,86,87,88]. Among the most extensively studied salivary exosomal miRNAs are miR-21, miR-155, miR-146a, and miR-223, which have been associated with periodontal inflammation and systemic inflammatory conditions. These molecules are increasingly recognized as promising non-invasive biomarkers for monitoring disease activity and evaluating the oral–systemic inflammatory axis [89,90].

### 3.9. Prenatal and Perinatal Influence

Prenatal and perinatal influence on epigenetics shows that the microbiota plays a role from the earliest stages of development [16]. It affects prenatal and perinatal epigenetics and contributes to the programming of the host’s immune, metabolic, and inflammatory responses. These effects can have long-term consequences on oral and systemic health, highlighting the importance of the microbial environment in biological development and epigenetic regulation even before birth and during the earliest moments of life.

**Figure 3:**
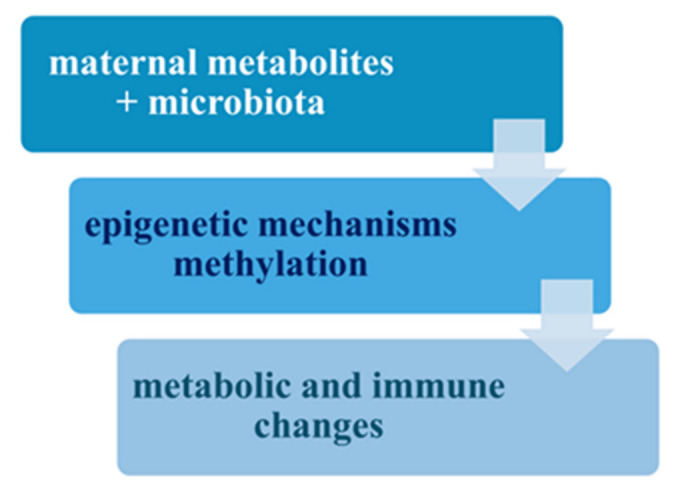
Impact of Maternal Microbiota and Metabolites on Epigenetic and Immune-Metabolic Outcomes.

Maternal microbiota and their metabolites influence epigenetic mechanisms, such as DNA methylation, leading to metabolic and immune changes in the host (Figure 3). Prenatal exposure to microbes and their products can train the fetal immune system through trained innate immunity and epigenetic mechanisms, influencing postnatal immune trajectories and the risk of asthma and allergies [91].

Further, previous studies demonstrated that the microbiota can influence host biology through epigenetic mechanisms, while other studies showed that it can support rapid physiological and functional adaptations without involving classical changes in host genomic DNA [11,19]. These findings are consistent with the concepts of the holobiont and hologenome, which emphasize the integrated functional interaction between host and associated microbial communities. In this context, it is important to clarify that microbial genes do not constitute a fixed proportion of “genetic material,” but rather represent a vastly expanded and dynamic gene repertoire at the metagenomic level, with microbial open reading frames (ORFs) outnumbering those of the human host by orders of magnitude.

Additionally, Joël de Rosnay et al. [92] emphasize the importance of DNA and RNA, including miRNAs, which constitute between 50% and 90% of epigenetic regulatory mechanisms, highlighting the complex ways in which the microbiota contributes to the development and adaptation of the organism even during prenatal and perinatal periods [92].

## 4. Discussions

This narrative review highlights that the oral cavity represents a biologically important host–microbiota interface, where structurally organized microbial communities interact with the host immune system and contribute to both oral and systemic health through complex metabolic and immunological interactions [4]. Rather than acting as an isolated ecosystem, the oral microbiome should be understood within a broader systemic network of host-microbe interactions [36]. One of the principal observations emerging from the reviewed literature is that the oral microbiome cannot be considered independently of the host. Rather, oral health is maintained through a dynamic network of host-microbial interactions, collectively referred to as the oralome [93]. Host tissues and microbial communities form a dynamic biological network in which immune regulation and metabolism are closely interconnected through bidirectional host–microbe interactions. In addition, microbiota-derived metabolites, particularly short-chain fatty acids such as butyrate, can influence host gene expression through epigenetic mechanisms, including histone deacetylase inhibition [94]. This integrated perspective supports the holobiont concept, although the extent to which the holobiont functions as a true unit of evolutionary selection remains a matter of scientific debate.

The evidence reviewed suggests that epigenetic regulation of host gene expression is mediated through mechanisms including DNA methylation, histone modifications, and non-coding RNAs, which collectively contribute to the regulation of cellular functions and disease processes. However, the majority of mechanistic data is derived from gut microbiome studies or experimental systems, and direct evidence within oral-specific contexts remains limited [95].

The types of cellular communication that maintain homeostasis involve the endocrine system through signaling molecules, the nervous system through neuronal impulses, and the immune system through specialized signaling molecules. In addition, some experimental studies have proposed that ultraweak photon emissions (biophotons) may represent a potential complementary mechanism of intercellular communication, although this hypothesis remains under investigation [42]. Nevertheless, current findings are predominantly associative, and causal mechanisms have not yet been fully established in human longitudinal studies. From an evolutionary perspective, the holobiont concept offers a unifying framework for understanding host–microbiome co-adaptation [12]. Within this context, early-life microbial exposure, beginning during the prenatal period through maternal microbial metabolites and continuing throughout the perinatal and early postnatal periods, contributes to immune system maturation and exerts long-lasting effects on host physiology [96]. However, these interpretations remain largely hypothetical and require further validation.

Clinically, the reviewed evidence supports the potential relevance of oral microbiome modulation as a preventive strategy. Approaches aimed at restoring microbial balance may contribute not only to oral health but also to systemic homeostasis. In addition, the use of salivary biomarkers may provide future opportunities for early disease detection [97]. Overall, the current literature supports a biologically plausible link between the oral microbiome and systemic regulation, but significant gaps remain in mechanistic understanding. Future research should focus on longitudinal human studies and multi-omics approaches to establish causal relationships and refine translational applications.

## 5. Therapeutic and Preventive Perspectives

Understanding the oral holobiont opens new therapeutic perspectives focused on microbiota modulation and epigenetic regulation. Preventive approaches include oral hygiene optimization, dietary interventions, probiotics, prebiotics, and postbiotics aimed at restoring microbial eubiosis [98,99,100]. In addition, salivary diagnostics and microbiome-based personalized medicine may contribute to the early detection and prevention of systemic diseases associated with oral dysbiosis [101]. Particular importance should be given to monitoring oral health during pregnancy, as maternal dysbiosis and inflammatory status may influence fetal epigenetic programming and long-term health outcomes.

## 6. Limitations

This narrative review has several limitations, as it is a narrative synthesis rather than a systematic or quantitative analysis, which may introduce selection bias and limits reproducibility. The included literature is highly heterogeneous, combining experimental and theoretical studies from different disciplines, which makes direct comparisons and causal interpretations difficult. In addition, several of the proposed mechanisms linking the oral microbiota to epigenetic regulation are still based on indirect evidence or extrapolations from gut microbiome research, and many concepts related to holobiont-level interactions and microbial-derived epigenetic signaling remain in an early stage of investigation. Therefore, further mechanistic and longitudinal studies are required to validate these associations and clarify the underlying biological pathways.

## 7. Conclusions

The evidence reviewed in this article highlights the oral cavity as a dynamic biological interface within the human holobiont, where continuous interactions between host tissues and the resident microbiota contribute to both local and systemic homeostasis. These interactions extend beyond classical microbial colonization and involve complex epigenetic mechanisms, including DNA methylation, histone modifications, microRNAs, extracellular vesicles, and microbial metabolites, which collectively regulate immune responses, tissue remodeling, and inflammatory pathways.

Oral dysbiosis may disrupt these regulatory mechanisms, promoting chronic inflammation and contributing to the development of systemic disorders through the oral–systemic axis. Increasing evidence also suggests that maternal oral microbiota and microbial metabolites may influence fetal immune programming and epigenetic regulation during prenatal and perinatal development, emphasizing the importance of maintaining oral health throughout pregnancy.

Future research should focus on integrating multi-omics approaches, longitudinal clinical studies, and mechanistic investigations to identify causal pathways and validate microbiome-derived biomarkers. Such advances may facilitate the development of personalized preventive and therapeutic strategies targeting the oral microbiome to improve both oral and systemic health.

## Figures and Tables

**Table 1:** Summary of key studies included in the narrative review.

Author (Year)	Focus Area	Study Type	Main Contribution	Relevance to Review
Rosenberg & Zilber-Rosenberg (2018) [10]	Holobiont/hologenome	Review	Defines holobiont and hologenome as a unit of selection	Core theoretical framework of holobiont
Bordenstein & Theis (2015) [1]	Holobiont principles	Review	Establishes ecological and evolutionary principles of holobionts	Conceptual foundation of host–microbiome integration
Henry et al. (2021) [11]	Microbiome and evolution	Review	Shows microbiome expands host evolutionary potential	Supports holobiont evolutionary model
Douglas & Werren (2016) [12]	Holobiont critique	Review	Critical evaluation of holobiont concept	Theoretical balance and conceptual debate
Gao et al. (2018) [2]	Oral microbiome	Review	Highlights importance of oral microbiome in systemic health	Foundation of oral microbiota relevance
Sedghi et al. (2021) [3]	Oral microbial ecology	Review	Describes microbial networks in oral health and disease	Supports oral ecosystem complexity and dysbiosis
Baker et al. (2024) [4]	Oral microbiome diversity	Review	Describes spatial and functional diversity of oral microbiota	Reinforces ecological structure of oral microbiome
Kilian et al. (2016) [13]	Oral microbiome update	Review	Clinical overview of oral microbiome and host interaction	Clinical relevance of oral microbiota
Hirschfeld & Kawai (2015) [14]	Oral-systemic inflammation	Review	Links oral bacteria with systemic inflammatory diseases	Key evidence for oral dysbiosis and inflammation axis
Woo & Alenghat (2022) [7]	Microbiota-epigenetics	Review	Describes epigenetic regulation via microbiota (DNA methylation, histones)	Core microbiota–epigenetic mechanism
Qin & Wade (2018) [15]	Microbiome-epigenome crosstalk	Review	Explains molecular communication between microbes and host epigenome	Theoretical framework for epigenetic regulation
Indrio et al. (2017) [16]	Early life microbiome programming	Review	Shows microbiome influence on immune and metabolic development	Supports prenatal/perinatal holobiont perspective

## Data Availability

No new data were created or analyzed in this study. Data sharing is not applicable to this article.
